# Surgical outcomes in patients with locally advanced gastric cancer treated with S-1 and oxaliplatin as neoadjuvant chemotherapy

**DOI:** 10.1186/s12957-015-0444-6

**Published:** 2015-01-30

**Authors:** Daofu Feng, Meiha Leong, Ting Li, Lin Chen, Tao Li

**Affiliations:** Department of General Surgery, General Hospital of Chinese PLA, Beijing, 100853 China; Department of General Surgery, General Hospital of Tianjin Medical University, Tianjin, 300052 China; Department of Surgery, Kiang Wu Hospital, Macao, China

**Keywords:** Gastric cancer, D2 lymphadenectomy, Pathological complete response, Complications, Neoadjuvant chemotherapy

## Abstract

**Background:**

We wished to evaluate the impact of S-1 combined with oxaliplatin (SOX regimen) as neoadjuvant chemotherapy on surgical outcomes after gastrectomy with D2 lymphadenectomy.

**Methods:**

From February 2012 to September 2013, 170 patients with American Joint Committee on Cancer (AJCC) stage II–III gastric cancer were assessed retrospectively. Eighty patients underwent neoadjuvant chemotherapy before radical gastrectomy, and 90 patients received surgical treatment with adjuvant chemotherapy. Patients received S-1 (80 mg/m^2^/day; days 1–14) and oxaliplatin (130 mg/m^2^; day 1) as neoadjuvant or adjuvant chemotherapy, and this schedule was repeated every 3 weeks. Gastrectomy with D2 lymphadenectomy was standard therapy for each patient. Surgical outcomes between the two groups were analyzed statistically.

**Results:**

There was no significant difference in the total prevalence of complications between neoadjuvant and adjuvant groups (18.8% vs. 22.2%, *P* = 0.704). The most common postoperative complications were surgical site infection (6.5%) and gastrointestinal motility disorders (3.5%). The clinical response rate was 68.8%, and ten patients (12.5%) had a pathological complete response after neoadjuvant chemotherapy. The SOX regimen as neoadjuvant chemotherapy for AJCC stage II/III gastric cancer can be effective without increasing the risk of postoperative complications.

**Conclusions:**

The SOX regimen could be a neoadjuvant chemotherapy for advanced gastric cancer worldwide in the future.

**Electronic supplementary material:**

The online version of this article (doi:10.1186/s12957-015-0444-6) contains supplementary material, which is available to authorized users.

## Background

Gastric cancer (GC) is the second leading cause of all cancer deaths worldwide [1,2] and a frequently diagnosed carcinoma in East Asia, Eastern Europe, and parts of South America [2-4]. For locally advanced GC, gastrectomy with D2 lymphadenectomy combined with adjuvant chemotherapy is the first-line treatment in Asia. S-1 (TS-1/Teysuno™) monotherapy for 1 year is used widely postoperatively in Japan [5]. However, this regimen is more appropriate for stage II disease rather than stage III disease [6]. Also, single-agent chemotherapy cannot be employed to treat advanced GC as standard chemotherapy [7]. Therefore, a more feasible and efficient doublet chemotherapy regimen based on S-1 needs to be developed for stage II or III patients in Asia. In contrast, other approaches have been established in Europe [8,9] and the United States [10].

Doublet chemotherapy using S-1 and cisplatin (SP) is the standard regimen for stage IV GC in Japan [11]. However, for locally advanced disease, cisplatin cannot be given during the first course of adjuvant chemotherapy after D2 surgery because grade 3 or 4 toxicities are observed quite frequently [12]. According to studies on chemotherapeutic drugs, it is shown that oxaliplatin is less toxic than cisplatin [13] and the S-1 plus oxaliplatin (SOX) regimen is not inferior to SP with regard to progression-free survival [14]. Therefore, the SOX regimen could be a novel option for locally advanced GC.

Since 2000, several phase III trials have not shown survival benefits for adjuvant chemotherapy, but perioperative chemotherapy was conducted in the MRC Adjuvant Gastric Infusional Chemotherapy (MAGIC) trial in the UK [8]. Owing to such positive results, neoadjuvant chemotherapy for locally advanced GC became a grade A recommendation in the guidelines for the management of GC by the National Comprehensive Cancer Network. Neoadjuvant chemotherapy has been accepted in some countries, but the optimal regimen has yet to be determined. Some patients cannot tolerate the toxicities induced by doublet or triplet chemotherapeutic drugs such as SP or ECF regimen after surgery. To increase the number of patients who complete treatment protocols for locally advanced GC, the SOX regimen should be preferred to the SP regimen because the former is less toxic and the efficacy of the two regimens should be identical.

Of course, adverse effects need to be considered, but postoperative complications cannot be ignored. The SOX regimen has low toxicity, but patients may not be able to undergo postoperative chemotherapy because of poor status induced by surgical complications. Therefore, to prove the safety of neoadjuvant chemotherapy with the SOX regimen, the morbidity and mortality after D2 surgery must be assessed.

The SOX regimen is as effective as cisplatin plus S-1 with favorable safety profile and is widely used for unresectable gastric cancer in Asia [15-17]. However, as for the SOX regimen, there is no randomized controlled trial (RCT) published for resectable gastric cancer. Thus, this protocol is a novel and potential treatment to prove the efficacy and safety for stage II and III gastric cancer.

In China, the SOX regimen is one of the first-line treatments for advanced GC [15]. The RCT on the SOX regimen neoadjuvantly is ongoing in our center [18]. Some specialized hospitals have participated in this program in China. However, the efficacy and safety of the SOX regimen in a neoadjuvant setting have not been well established. Here, we wished to assess the tumor responses and postoperative morbidity for patients receiving perioperative SOX chemotherapy compared with adjuvant chemotherapy.

The objective of this study is to determine whether neoadjuvant chemotherapy with the SOX regimen in stage II and III patients could make radical surgery feasible and improve the prognosis.

## Methods

### Ethical approval of the study protocol

The study protocol was approved by the Ethics Committee of the Chinese PLA General Hospital (Beijing, China). All patients provided informed consent for the procedure in the study.

### Study design

**Figure 1:**
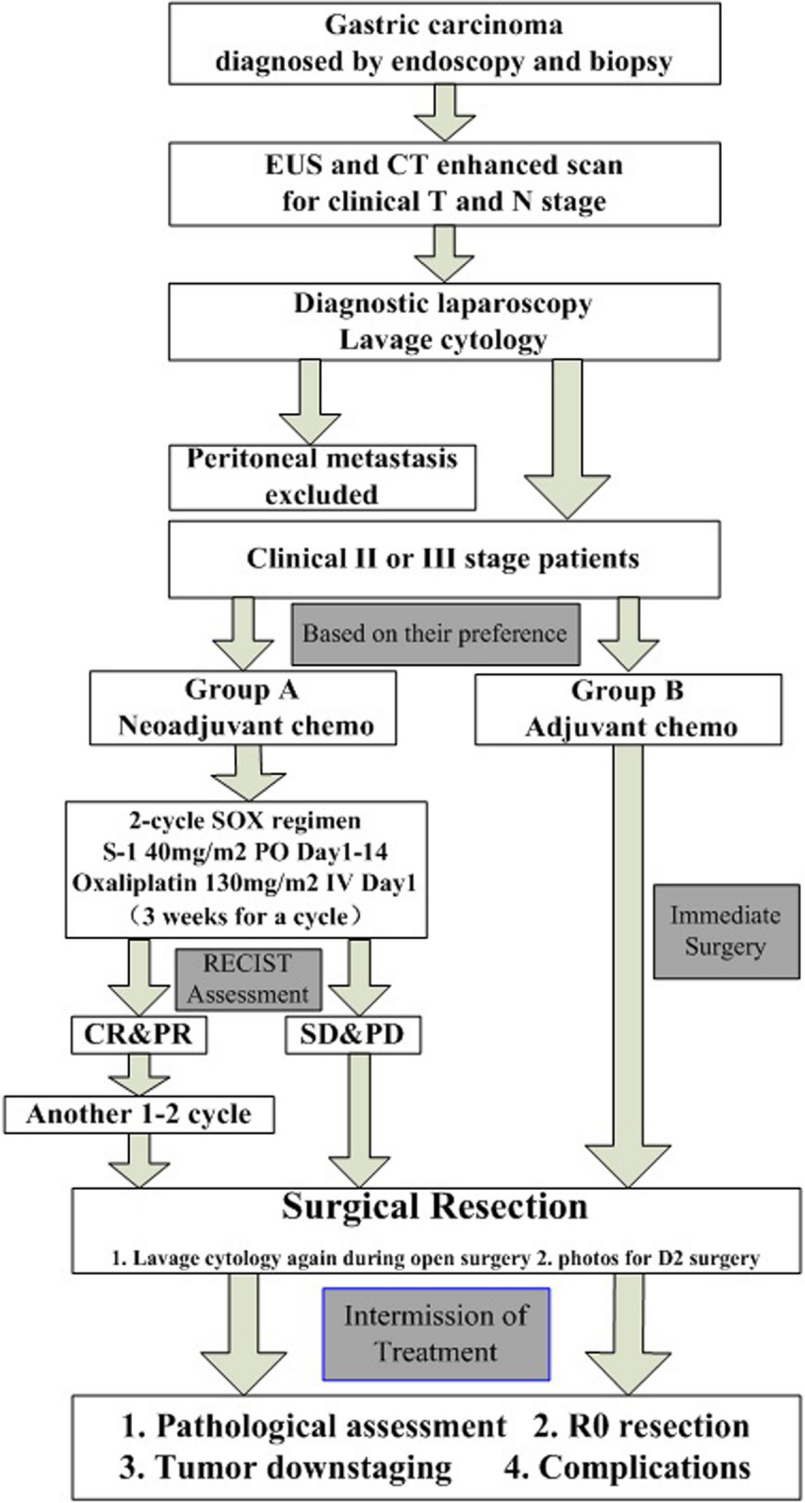
**Study design.**

Gastric carcinoma was diagnosed by endoscopy and biopsy. Endoscopic ultrasound (EUS) and enhanced CT scan were operated for clinical T and N stages. Diagnostic laparoscopy and lavage cytology were performed to exclude peritoneal metastasis. After clinical staging, stage II or III patients were selected and assigned to two groups based on their preference. Group A received two-cycle SOX regimen and evaluated by Response Evaluation Criteria in Solid Tumors (RECIST) 1.1. If the assessment is complete response (CR) or partial response (PR), the patients should be given another one or two cycles. If the assessment is stable disease (SD) or progressive disease (PD), the surgery need be performed directly. In group B, surgery should be operated immediately. After surgery, tumor staging, pathological results, R0 resection, and complications were evaluated to prove the efficacy and safety of the SOX regimen as neoadjuvant chemotherapy (Figure 1).

### Patients

This retrospective study initially consisted of 201 patients with GC who underwent resection from the Chinese PLA General Hospital from February 2012 to September 2013. All the clinicopathological information was available, including demographic data, patients’ comorbidities, surgical parameters, image study information, pathological diagnosis, perioperative therapies, tumor response, and follow-up data. Among these patients with GC, only those who had stage II–III GC and D2 lymphadenectomy were enrolled into the study. Patients who had D0 or D1 resection, para-aortic lymph node involvement, Virchow lymph node metastasis, and incomplete neoadjuvant chemotherapy were excluded because this study mainly focused on those patients under D2 resection. Finally, 170 patients were enrolled into this study.

Of these 170 individuals, 80 patients received SOX neoadjuvant chemotherapy and 90 patients received surgical treatment with adjuvant chemotherapy. Patients had been proven to have GC by histological analyses. Subjects had been assessed clinically as being T2–4, N0–3, and M0 according to the third English edition of the Japanese Classification of Gastric Carcinoma [19], and tumor size measured according to RECIST v1.1 [20]. Additional eligibility criteria comprised no distant/peritoneal metastases and negative cytology by contrast-enhanced computed tomography (CE-CT) and laparoscopy, as well as Eastern Cooperative Oncology Group (ECOG) performance status (PS) of 0 or 1. Patients had not received chemotherapy or radiotherapy previously. The clinical diagnosis of T and N stages was based mainly on EUS and CE-CT. Diagnostic laparoscopy and lavage cytology were used to exclude peritoneal metastasis by following the method of Yano et al. [21]. In order to improve the accuracy of clinical diagnosis, all patients underwent laparoscopic examination. Therefore, all patients were clinically diagnosed as stage II or III based on ultrasonic endoscopy, CE-CT scan, and staging laparoscopy. Patients were assigned to each arm of the study based on their preference after detailed explanation of the advantages and disadvantages of the two regimens. Meanwhile, patients provided informed consent to receive the corresponding treatment. After the treatment protocols, the data were analyzed and summarized following these 170 cases retrospectively. Patients who underwent emergency surgery and who did not undergo D2 resection were excluded from the study. D2 surgery was the standard treatment in Asia. To prolong the overall survival, we performed D2 surgery routinely. If patients had severe cardiac disease, significant cerebrovascular illness, or pregnancy, the treatment protocol cannot be used. Similarly, patients with another diagnosed malignancy in recent 5 years were precluded. Therefore, 170 patients that meet study criteria during the stated time period were included (Additional files 1 and 2).

### Preoperative chemotherapy

S-1 was administered via the oral route twice a day for a total of 80 mg/m^2^ from day 1 to day 14, and oxaliplatin was given as an intravenous infusion of 130 mg/m^2^ on day 1. This regimen was repeated every 3 weeks for two to four cycles. Blood tests were carried out, blood biochemistry assessed, levels of tumor markers [carcinoembryonic antigen (CEA), cancer antigen (CA) 19–9] measured, physical examination undertaken, and PS checked <1 week before the initiation of chemotherapy. Blood tests were carried out, symptoms evaluated, and physical examination carried out each week during treatment. The regimen was modified by decreasing S-1 from 120 to 100 or 80 mg/day, and oxaliplatin from 130 to 100 or 85 mg/m^2^ if patients had a white blood cell (WBC) count ≤4,000/mm^3^, platelet count ≤10,000/mm^3^, abnormal feelings in peripheral nerves, diarrhea, or nausea of grade ≥2. If adverse events worsened, the regimen was postponed until recovery. After two cycles of chemotherapy, tumor response was evaluated based on the findings of CE-CT and EUS according to RECIST 1.1 [20]. If tumors had increased in size, resection was carried out immediately. If not, one or two additional cycles of chemotherapy were given before D2 surgery.

### Surgery and postoperative chemotherapy

Patients underwent surgical resection within 6 weeks after completion of SOX neoadjuvant chemotherapy. For all patients, gastrectomy with D2 lymphadenectomy was conducted by experienced surgeons according to criteria set by the Japanese Gastric Cancer Association [19]. Intraperitoneal wash cytology was carried out at the beginning of each surgical procedure. Based on the location of the primary tumor, distal, proximal, or total gastrectomy was carried out. In the neoadjuvant chemotherapy group, patients underwent four to six cycles of chemotherapy with the SOX regimen after surgery. Patients in the other group received eight cycles of adjuvant chemotherapy with the SOX regimen.

### Complications

Complications were recorded prospectively for each patient after surgery. Surgery-related complications included surgical site infection, postoperative hemorrhage, anastomotic leakage, ileus or bowel obstruction, wound dehiscence, biliary fistulae, and lymphatic fistulae. Non-surgical complications were gastrointestinal motility disorders or gastroplegia, pulmonary infection, thrombocytopenia, catheter-related sepsis, thrombosis, and renal dysfunction.

### Pathological assessment

Resection specimens were examined by the same experienced pathologist. Postoperative pathology was reported as follows: (i) Lauren pathological classification for primary tumors; (ii) histologic response was assessed according to the proportion of tumor affected by degeneration or necrosis [19]; (iii) resection margins were classified as R0 (no cancer at the resection margin), R1 (microscopically involved margin), and R2 (macroscopically involved margin); and (iv) positive and negative lymph nodes in each group.

### Statistical analyses

Data were collected and supervised by the same surgeon. Results are the median or means for continuous variables depending on the one-sample Kolmogorov-Smirnov test (by which normal distribution was determined). Proportions are expressed as qualitative variables. Means, median, and proportions between the two groups were compared using the Student’s *t* test, Mann–Whitney *U* test, or Pearson chi-square test. Relative analysis was performed by ordinal multinomial logistic regression. *P* ≤ 0.05 (two-sided) was considered significant. Statistical analyses were conducted using SPSS v19.0 (SPSS, Chicago, IL, USA).

## Results

### Patient characteristics

One-hundred and seventy patients were assigned to the neoadjuvant group (*n* = 80) or adjuvant group (*n* = 90). The median age of the study cohort was 60 years (range, 21–82). The two groups were similar in terms of age, sex, and body mass index (BMI). The distribution was also well balanced with respect to tumor location and clinical TNM staging (Table 1). The PS of patients in the two groups according to the ECOG was 0 or 1.

**Table 1:** **Patient characteristics**

	**Neoadjuvant (** ***n*** **= 80)**	**Adjuvant (** ***n*** **= 90)**	***P***
Age (years)	60 (21–74)	59 (29–82)	0.762
Sex			0.982
Male	63 (78.8%)	71 (78.9%)	
Female	17 (21.2%)	19 (21.1%)	
BMI (kg/m^2^)	23.8	23.6	0.709
> 25	26 (32.5%)	29 (32.2%)	
Tumor location			0.135
GEJ	32 (40.0%)	21 (23.3%)	
Body	15 (18.8%)	22 (24.5%)	
Distal	32 (40.0%)	45 (50.0%)	
Total	1 (1.2%)	2 (2.2%)	
Clinical TNM stage			0.172
II	4	10	
III	76	80	

*BMI* body mass index, *GEJ* gastroesophageal junction.

### Efficacy of neoadjuvant treatment

Patients in the neoadjuvant group received at least two cycles of preoperative SOX chemotherapy. Thirteen (16.3%), 18 (22.5%), and 49 patients (61.3%) received two, three, and four cycles, respectively. Ten American Joint Committee on Cancer (AJCC) II–III patients (12.5%) achieved a CR, and 45 patients (56.3%) received a PR. Twenty-three patients (28.7%) had SD and two patients (2.5%) suffered from PD, resulting in an overall response rate (RR) of 68.8% [95% confidence interval (CI) 0.58–0.79] and disease control rate of 97.5% (95% CI 0.94–1.0) (Table 2). Toxicities induced by neoadjuvant chemotherapy were well tolerated and manageable in all cases.

**Table 2:** **Clinical response after SOX neoadjuvant chemotherapy**

**Response**	**No. of patients**	**% (95% CI)**
CR	10	12.5
PR	45	56.3
SD	23	28.7
PD	2	2.5
Overall response rate	55	68.8 (58.6–79.0)
Disease control rate	78	97.5 (94.1–100)

*CI* confidence interval, *CR* complete response, *PR* partial response, *SD* stable disease, *PD* progressive disease. Disease control rate = CR + PR + SD.

The results of the responses in the NAC group associated with tumor locations in the stomach are summarized in Table 3. However, the clinical responses were irrelevant to tumor locations by ordinal multinomial logistic regression (*P* = 0.873).

**Table 3:** **Relative analysis on clinical responses**

	**No.**	**CR**	**PR**	**SD**	**PD**	***P***
GEJ	32	4	20	7	1	
Body	15	1	9	5	0	0.543
Distal	32	5	15	11	1	0.504
Total	1	0	1	0	0	0.845

*GEJ* gastroesophageal junction, *CR* complete response, *PR* partial response, *SD* stable disease, *PD* progressive disease.

### Preoperative status

WBC counts and platelet counts were lower in the neoadjuvant group than in the adjuvant group (*P* < 0.001). This result was in accordance with the most common grade 3/4 toxicities (neutropenia and thrombocytopenia) observed in the SOX regimen [14]. CEA or CA 19–9 represents a strong prognostic factor for GC (especially in patients with high preoperative levels of these markers), so measurement of these tumor markers before surgery was essential [22]. There was no significant difference in preoperative levels of CEA and CA 19–9 between the two groups. Before the neoadjuvant chemotherapy, the median maximum diameter of tumors observed by EUS was similar in both groups (5 vs. 5 cm, *P* = 0.329). Both groups were well balanced in terms of hemoglobin levels, serum levels of albumin, comorbidity, and tumor size (Table 4).

**Table 4:** **Preoperative status**

	**Neoadjuvant (** ***n*** **= 80)**	**Adjuvant (** ***n*** **= 90)**	***P***
White blood cells	4.73	5.63	<0.001
Platelets	156.7	240.4	<0.001
Hemoglobin	125.1	121.5	0.291
Serum albumin	39.4	38.9	0.520
CEA >5 μg/L	17 (21.8%)	14 (16.3%)	0.427
CA 19–9 > 37 μg/mL	13 (16.7%)	16 (18.6%)	0.839
Comorbidity	43 (53.8%)	54 (60.0%)	0.441
Cardiovascular	31 (38.8%)	28 (31.1%)	0.335
Pulmonary	3 (3.8%)	5 (5.6%)	0.724
Diabetes mellitus	2 (2.5%)	9 (10.0%)	0.062
Gastrointestinal disease	7 (8.8%)	4 (4.4%)	0.352
Urinary	4 (5.0%)	1 (1.1%)	0.189
Liver	4 (5.0%)	8 (8.9%)	0.381
History of surgery	22 (27.5%)	21 (23.3%)	0.597
Other	12 (15.0%)	20 (22.2%)	0.245

*CA 19–9* cancer antigen 19–9, *CEA* carcinoembryonic antigen.

### Operative details

The mean duration of surgery was 230 min in the neoadjuvant group and 212 min in the adjuvant group (*P* = 0.017). As a result of hemorrhage or tissue edema induced by neoadjuvant chemotherapy, the operative time was increased to ensure careful resection. The median operative blood loss in the neoadjuvant group was 300 mL (range, 100–700), which was significantly different from that of the adjuvant group (*P* = 0.05). The volume of blood loss during the operation was much lower in the adjuvant group. In the neoadjuvant group, four patients (5%) were transferred to the intensive care unit and 12 (15%) received a blood transfusion during surgery. There was no difference in terms of type of resection or reconstruction (Table 5). D2 lymphadenectomy was conducted for each patient, and the number of harvested lymph nodes in the two groups was not significantly different (29 vs. 30, *P* = 0.317). Splenectomy was carried out in three patients (3.3%) in the adjuvant group. Resection was curative in 76 patients (95.0%) in the neoadjuvant group compared with 85 subjects (94.4%) who received adjuvant chemotherapy after surgery (*P* = 0.872).

**Table 5:** **Surgical results**

	**Neoadjuvant (** ***n*** **= 80)**	**Adjuvant (** ***n*** **= 90)**	***P*** ^**a**^
Total operative time	230.4	212.2	0.017
Operative blood loss (mL)	300	200	0.050
≥ 200	66 (83.5%)	60 (66.7%)	0.014
Procedure			0.320^b^
Total gastrectomy	23 (28.8%)	30 (33.3%)	0.249^c^
Roux-en-Y	23 (100.0%)	27 (90.0%)	
Braun	0 (0%)	3 (10.0%)	
Distal gastrectomy	33 (41.2%)	42 (46.7%)	0.425^d^
Billroth I	26 (78.8%)	28 (66.7%)	
Billroth II	2 (6.1%)	6 (14.3%)	
Roux-en-Y	5 (15.1%)	8 (19.0%)	
Proximal gastrectomy	24 (30.0%)	18 (20.0%)	—
Esophagogastric anastomosis	24 (100.0%)	18 (100.0%)	
Extent of resection			0.872
R0	76 (95.0%)	85 (94.4%)	
R1/R2	4 (5.0%)	5 (5.6%)	
Mean no. of nodes removed	29	30	0.317

^a^Chi-square test; ^b^comparison of total, distal, and proximal gastrectomies; ^c^comparison of Roux-en-Y and Braun; ^d^comparison of Billroth I, Billroth II, and Roux-*en*-Y.

### Pathological findings

The median maximum diameter of tumors obtained from pathological specimens was smaller in the neoadjuvant group than in the adjuvant group (2.5 vs. 5 cm, *P* < 0.001), a finding in accordance with tumor shrinkage induced by preoperative chemotherapy. Ten patients (12.5%) had a pathological complete response (pCR), whereas two other patients in the neoadjuvant group were evaluated as ypT0N1M0. Before the neoadjuvant chemotherapy, the clinical T staging was not significantly different in both groups (*P* = 0.323). After the surgical resections, the pathological results showed that there was a smaller proportion of stage T3 and T4 tumors in the neoadjuvant group than in the adjuvant group (43.8% vs. 90.0%, *P* < 0.001). Fifty-six patients (70%) had tumor downstaging. An obvious trend of less metastasis of lymph nodes in the neoadjuvant group than in the adjuvant group was noted (58.8% vs. 14.4%, *P* < 0.001) (Table 6).

**Table 6:** **Pathological results**

	**Neoadjuvant (** ***n*** **= 80)**	**Adjuvant (** ***n*** **= 90)**	***P***
Clinical/pathological tumor stage			0.323/<0.001
T0	0 (0%)/12 (15.0%)	0 (0%)/0 (0%)	
T1	4 (5.0%)/9 (11.2%)	1 (1.1%)/1 (1.1%)	
T2	7 (8.8%)/24 (30.0%)	10 (11.1%)/8 (8.9%)	
T3	16 (20.0%)/15 (18.8%)	13 (14.4%)/16 (17.8%)	
T4	53 (66.3%)/20 (25.0%)	66 (73.4%)/65 (72.2%)	
Nodal status			**<**0.001
N0	47 (58.8%)	13 (14.4%)	
N1 (one to two nodes involved)	13 (16.3%)	20 (22.2%)	
N2 (three to six nodes involved)	11 (13.8%)	25 (27.8%)	
N3 (more than seven nodes involved)	9 (11.3%)	32 (35.6%)	

### Complications

The overall morbidity was 20.6% and not significantly different between the two groups (18.8% vs. 22.2%, *P* = 0.704). Surgical and non-surgical complications are listed in Table 7. Gastrointestinal motility disorders and surgical site infection were the most common complications. Postoperative hemorrhage was more common in the neoadjuvant group, whereas anastomotic leakage and pulmonary problems were more common in the adjuvant group. Pancreatic fistulae or abdominal abscesses were not observed in either group. No patients required reoperation and none of our patients died.

**Table 7:** **Postoperative morbidity and mortality**

	**Neoadjuvant (** ***n*** **= 80)**	**Adjuvant (** ***n*** **= 90)**	***P***
PHS (days)	11 (7–33)	11 (5–51)	0.920^a^
PHS with complications (days)	15	17	0.503^b^
PHS without complications (days)	11	11	0.972^a^
Patients with complications	15 (18.8%)	20 (22.2%)	0.704
Non-surgical complications			
Pneumonia	1 (1.3%)	3 (3.3%)	0.623
Pleural effusion	0 (0.0%)	2 (2.2%)	0.499
Gastrointestinal motility disorders	3 (3.8%)	3 (3.3%)	1.000
Thrombosis	1 (1.3%)	1 (1.1%)	1.000
Renal dysfunction	1 (1.3%)	1 (1.1%)	1.000
Catheter-related sepsis	1 (1.3%)	0 (0.0%)	0.471
Other (urinary, thrombocytopenia)	1 (1.3%)	4 (4.4%)	0.372
Surgical complications			
Anastomotic leakage	0 (0.0%)	2 (2.2%)	0.499
Surgical site infection	5 (6.2%)	6 (6.7%)	1.000
Postoperative hemorrhage	3 (3.8%)	1 (1.1%)	0.343
Wound dehiscence	0 (0.0%)	2 (2.2%)	0.499
Lymphorrhea	2 (2.5%)	1 (1.1%)	0.602
Ileus	0 (0.0%)	1 (1.1%)	1.000
Biliary fistula	1 (1.3%)	0 (0.0%)	0.471

*PHS* postoperative hospital stay. ^a^Statistical analyses by the Mann–Whitney *U* test due to non-normal distribution in one or both groups; ^b^statistical analyses by the Student’s *t* test due to a normal distribution in both groups.

The overall median postoperative stay in the hospital was 11 days in both groups (*P* = 0.920). For patients suffering complications, the mean postoperative stay in the hospital was 15 days in the neoadjuvant group and 17 days in the adjuvant group (*P* = 0.503). A postoperative stay was much shorter if complications did not occur.

## Discussion

The prevalence of postoperative complications after neoadjuvant chemotherapy has been reported to range from 10% to 46% and is based on the experience of the operating surgeon, multi-visceral resections, extended lymph node dissections, different regimens, and elderly patients with comorbidities [8,23-25]. As for neoadjuvant treatments, some surgeons still believe that preoperative chemotherapy may induce many early complications after D2 surgery for locally advanced GC. Studies assessing the impact of the SOX regimen as neoadjuvant chemotherapy for AJCC stage II–III GC on surgical morbidity and mortality are also lacking. Therefore, the assessment on the safety of SOX neoadjuvant chemotherapy for locally advanced GC is essential.

In the present study, the prevalence of postoperative morbidity in the neoadjuvant group was 18.8%. This result is in accordance with reports on the prevalence of complications in patients who received D2 surgery without preoperative chemotherapy [26-28]. Those data were comparable to our results, in which postoperative morbidity was seen in 17.9–29.4% of subjects. Relaparotomy for surgical complications was necessary in the Dutch gastric cancer trial [29]. Anastomotic leakage and pancreatic fistulae were the most common complications for reoperations. Fortunately, no patient in the current series underwent reoperations. Pancreatic fistulae were not seen in the study. The single leakage was managed by jejunal feedings, nasogastric tube, abdominal tube drainage, and antibiotics. Pancreatic fistulae and anastomotic leakage should be observed frequently for D3 surgery or multi-visceral resections. These results could be attributed to modern surgical devices, experienced surgeons, and prophylactic application of octreotide acetate after D2 surgery. Infection at the incision site and gastrointestinal motility disorders (especially gastroplegia) were observed frequently in the present study, but these complications are minor and managed easily. For surgical site infections, the bacteria need to be found, drug susceptibility test has to be conducted, antibiotics need to be utilized, and dressings have to be changed. In terms of postoperative hemorrhage, adequate blood transfusion and hemocoagulase were essential and effective. Therefore, we agree with the opinion that complications should be managed conservatively and reexploration reserved for patients whose conservative treatment is failed [30]. Multi-visceral resections, especially splenectomy and distal pancreatectomy, were rarely needed. The Dutch trial had reported that extended lymphadenectomy combined with multi-visceral resection should be limited due to the increased risk of surgery-related mortality and morbidity [29]. If the tumor directly extended to the spleen, transverse colon, or tail of pancreas, multi-visceral resections need to be performed to achieve a high R0 rate. In this study, the low rate of deaths and complications may be associated with limited multi-visceral resections. In the MAGIC trial, the prevalence of morbidity in the chemotherapy group was 45.7% [8]. The SOX regimen is less toxic than the triplet regimen, so surgical morbidity does not seem to increase even after D2 lymphadenectomy.

Because of fibrosis and tissue edema resulting from chemotherapy, D2 lymphadenectomy after neoadjuvant chemotherapy seemed to be more risky than D2 lymphadenectomy without preoperative treatment. Operative hemorrhage should be easily induced by fibrosis and tissue edema, although an ultrasonic knife plays an important role in hemostasis. Therefore, more operative blood loss can be seen in the neoadjuvant group. Indirectly, it took harder and longer to stop the bleeding (Table 5). However, no deaths occurred during and after D2 surgery upon neoadjuvant chemotherapy with the SOX regimen. In a phase II trial of neoadjuvant chemotherapy with the SP regimen, surgical mortality was not observed [24]. Another phase II trial was conducted to assess the safety of neoadjuvant chemotherapy with irinotecan and cisplatin before D3 resection but was terminated, and the prevalence of surgical mortality was nearly 2.0% [31].

The number of courses has not been determined so far. We arranged the courses based on MAGIC, FFCD, and COMPASS trials which demonstrated that three or four neoadjuvant courses could achieve a better prognosis [8,9,32]. Due to the main advantage of neoadjuvant chemotherapy which is good tolerance of preoperative treatment, patients receiving more than two cycles of chemotherapy did not have grade 3 or 4 adverse outcomes. Less toxic oxaliplatin may also contribute to the result [16]. Thus, if the patients were not strongly willing to perform the operation immediately, we recommended three or four courses to them.

Clinical responses in 31 patients were evaluated as SD or PD when the criteria were analyzed after two cycles. In these patients, the responses of 13 patients who received two cycles were not sensitive to the SOX regimen. Thus, D2 surgery was performed for them immediately. On the other hand, the response of the other 18 patients was mildly sensitive to SOX chemotherapy but not evaluated as PR after two courses. Therefore, following the recommended number of courses used by the MAGIC trial, we conducted one more cycle for them. In these 18 patients, the response of a few patients was assessed as PR before surgery because of relatively adequate courses. Disease control rate was 97.5%. Thus, we considered that this treatment protocol did not worsen the situation in these patients.

The present study demonstrated a relatively high clinical RR of 68.8%, which was virtually the same as that observed for tumor downstaging. In three phase II trials using the SOX regimen against advanced GC, the clinical RRs were 55.3%, 59.0%, and 53.7% [14,33,34]. The RR was higher because 67 (83.8%) patients received three or four cycles of preoperative chemotherapy in the present study. The COMPASS trial proved that patients benefitted from adequate courses of neoadjuvant chemotherapy, which was consistent with this study [32].

All patients received D2 surgical resections with good tolerance of SOX neoadjuvant chemotherapy, while Kochi reported that 88% eligible patients underwent curative surgery with SP regimen as neoadjuvant chemotherapy [35]. This result may contribute to a less toxic SOX regimen because no patient had grade 3 or 4 toxicities in this study.

Before the initiation of preoperative chemotherapy, the clinical T staging showed no significant difference in the two groups (*P* = 0.323). However, after the patients underwent D2 resection, the pathological results demonstrated that the proportion of stage T3 and T4 tumors in the neoadjuvant group was smaller than in the adjuvant group. There was also less metastasis of lymph nodes in the neoadjuvant group than in the adjuvant group. The downstaging rate and pathological response were consistent with clinical response in the neoadjuvant group. Furthermore, the tumors were obviously shrunk after neoadjuvant chemotherapy. These results were attributed to the efficacy of SOX neoadjuvant chemotherapy.

A pCR is associated with prognosis, so the primary endpoint was pCR in this study [36]. Thus, all the patients received D2 surgery. In the neoadjuvant group, only two patients were performed the surgery immediately when the assessment was PD. Another major finding of the present study was a high pCR of 12.5% after neoadjuvant chemotherapy with the SOX regimen. Several studies have failed to show that preoperative chemotherapy can elicit such high pCRs [8,9,23-25,31,32]. Furthermore, in the ten patients who achieved a pCR, nine patients received three or four cycles of the SOX regimen, which was in accordance with the results of the COMPASS trial [32]. These results suggested that the SOX regimen was a promising treatment to achieve high pCRs with an adequate number of courses.

This retrospective study compared the results of two groups at the same institution. There was a good balance in each group in terms of the characteristics of patients and tumors, but patients were enrolled based on their preference and treated with at least two courses depending on the sensitivity to neoadjuvant chemotherapy. Surgeon experience and the interpretations of the pathologist could lead to a confounding bias, so the procedures were carried out by the same team of surgeons, and resections assessed by the same experienced pathologist. This trial did not include node-negative advanced gastric cancer in the neoadjuvant group, which may be attributed to inevitably overdiagnosed preoperative staging and the relatively critical illness in our center. In the present analysis, we mainly focused on the patients with lymph node metastasis stage II–III gastric cancer.

## Conclusions

Here, we showed that neoadjuvant chemotherapy with at least two cycles of the SOX regimen could induce a relatively high number of pathological CRs without increasing the prevalence of morbidity and mortality compared with D2 resection alone. In this regard, neoadjuvant chemotherapy with the SOX regimen followed by gastrectomy with D2 resection is an effective and feasible candidate for treating AJCC stage II/III GC. A prospective RCT to evaluate the efficacy and survival benefits of neoadjuvant chemotherapy with the SOX regimen for locally advanced GC has been launched in China (RESONANCE; clinical trial number NCT01583361).

## Additional files

Additional file 1:
**Basic characteristics for each patient.**
Additional file 2:
**Comprehensive descriptions for the two groups.**
